# Identification of two novel homozygous nonsense mutations in *TRAPPC9* in two unrelated consanguineous families with intellectual Disability from Iran

**DOI:** 10.1002/mgg3.1610

**Published:** 2021-01-29

**Authors:** Farideh Yousefipour, Hossein Mozhdehipanah, Frouzandeh Mahjoubi

**Affiliations:** ^1^ National Institute of Genetic Engineering and Biotechnology Tehran Iran; ^2^ Department of Neurology Bou Ali Sina Hospital Qazvin University of Medical Sciences Qazvin Iran

**Keywords:** intellectual disability, Iran, nonsense mutation, *TRAPPC9*

## Abstract

**Background:**

Pathogenic mutations in *TRAPPC9* are associated with autosomal recessive Intellectual Disability (ID), a major public health issue that affects about 1–3% of children worldwide.

**Method:**

Clinical evaluation, magnetic resonance imaging, peripheral blood karyotype, Multiplex ligation‐dependent probe amplification (MLPA), array CGH, and whole‐exome sequencing were used to characterize etiology in three patients from two unrelated consanguineous families of Iranian descent with intellectual disability.

**Results:**

Whole‐exome sequencing showed two novel homozygous nonsense mutations (c.937C>T) in exon 3 and (c.3103C>T) in exon 19 of *TRAPPC9* (NM_031466.7) in two unrelated consanguineous families.

**Conclusion:**

The two novel variants found in *TRAPPC9* caused truncated protein and clinical manifestations such as ID, developmental delay, microcephaly, and brain abnormalities in three patients.

## Graphical abstract

We describe three patients with intellectual disability , born to two healthy and consanguineous families in whom two novel homozygous nonsense mutations of TRAPPC9 gene were identified by whole exome sequencing.

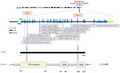

## 1 INTRODUCTION

Intellectual disability (ID) is a neurodevelopmental disorder, characterized by considerable limitation of intellectual functioning, adaptive behavior, or daily living skills, and with an onset before 18 years of age (Heidari et al., 2015). It is one of the most important challenges in health care, with significant life‐long socioeconomic burden. ID is genetically heterogeneous and may result from chromosomal aberrations, or from either autosomal recessive (AR), autosomal dominant, X‐linked, or mitochondrial mutations. With the prevalence of ∼1% of children worldwide (Maulik et al., 2011; Musante & Ropers, 2014), ID can be divided into two main groups: nonsyndromic (NS) ID, where it might display the sole clinical feature, and syndromic ID, where additional clinical or dysmorphological features may also be present. Over the past few years, next‐generation sequencing technologies have led to the identification of a number of ID‐associated genes, emphasizing the considerable genetic heterogeneity of ID.

Trafficking protein particle complex subunit 9 gene (*TRAPPC9*; OMIM# 611966) plays an important role in the neuronal NF‐kB signaling pathways and is one of the numerous genes involved in the nonsyndromic form of ID (MIM 611966). Pathogenic homozygous mutations of *TRAPPC9* have been reported to cause autosomal recessive ID (IDT 13, MIM#613192). Patients with *TRAPPC9* mutations in previous studies displayed intellectual disability, developmental delay, microcephaly, and brain abnormalities (Abbasi et al., 2017; Jamra et al., 2011; Bai & Kong, 2019; Giorgio et al., 2016; Hnoonual et al., 2019; Kakar et al., 2012; Marangi et al., 2013; Mir et al., 2009; Mochida et al., 2009; Mortreux et al., 2018; Philippe et al., 2009).

Here, we describe three patients with ID, born to healthy and consanguineous families in whom two novel homozygous nonsense mutations of *TRAPPC9* were identified by whole‐exome sequencing.

### 1.1 Patient data

In this study, we report three patients with clinical features compatible with intellectual disability, born to two unrelated consanguineous families in Iran, with no family history of similar findings. Family members, including the affected individuals, were clinically assessed by an experienced neurologist. The prenatal, perinatal, and neonatal medical histories of the patients were normal.

### 1.2 Patient 1

**FIGURE 1:**
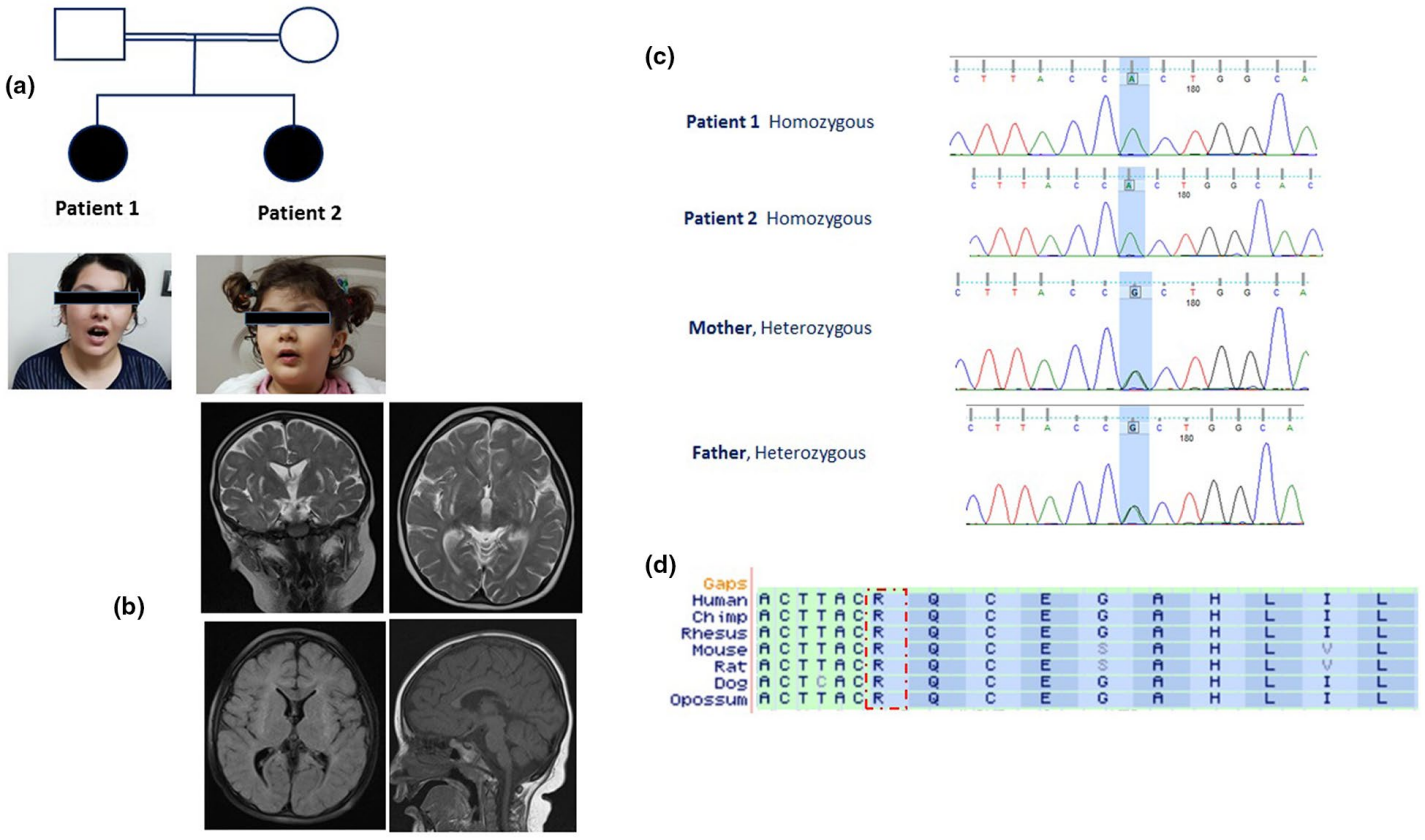
(Analysis of family 1; a) Pedigree of the consanguineous family 1 with two patients affected by intellectual disability without microcephaly. (b) Brain MRI of patient 2 reveals global brain atrophy in the white matter and thinning of corpus callosum. (c) Electropherograms from Sanger confirmation in family members showing NM_031466.7 (*TRAPPC9*:c.937C>T; p.Gln313Ter.) Heterozygous and homozygous sequence. (d) ClustalW2 (http://www.ebi.ac.uk/Tools /msa/clustalw2) alignment/comparison of *TRAPPC9* across vertebrate species showing conservation at p.Gln313 (red rectangle).

Patient 1 (Figure 1) was a 16‐year‐old girl, born via normal vaginal delivery to a healthy consanguineous family, with normal growth parameters after an unremarkable pregnancy and delivery. Developmental delay became obvious by the second year and after therapeutic intervention, she began walking around 24 months of age, but a significant speech delay was noted thereafter. At age 7, she was diagnosed with ID and Attention Deficit Hyperactivity Disorder (ADHD) as well as aggressive behavior and self‐injury (cutting, scratching, and hitting body parts). At age 7, her occipitofrontal circumferences (OFC) was 48.5 cm (−2SD). At the time of examination, OFC was 54.5 cm (50th percentile). Her nonverbal IQ was 30–40 (sever impairment). She had severe cognitive delay and her language was limited to only a few single words. Behavior and social evaluation showed that she had sever deficit in adaptive behaviors in all domains including communication, daily living skills, and socialization and she was not able to perform any of her activities of daily living independently. She also showed agitation and restlessness, manifested by pacing, hand‐wringing, and fidgeting. The gait was normal and she walked with a wide base. However, she was not able to sit and pull herself up without assistance. She had normal muscle tone with no spasticity and except for unequivocal visuospatial perception, her neurological examination was normal. She also demonstrated flexion contracture of elbow and fingers without history of trauma and immobilization. Brain magnetic resonance imaging (MRI) was performed for the patient and showed a thin but fully formed corpus callosum but MRI image was not available. The height and weight were within the normal range as compared to the local population. No history of seizures or regression has been reported and there were no autistic features as well. Routine blood hematology and biochemistry tests were within the normal range for the patient.

### 1.3 Patient 2

Patient 2 (Figure 1) was a 5‐year‐old girl and the second child of family 1 who was born via normal vaginal delivery. Nothing remarkable was noticed during pregnancy nor during the first months of life and growth parameters were normal. Head circumference at birth was 33 cm (15th percentile). The birth weight was 3.7 kg (85th percentile) and the birth height was 49 cm (50th percentile). Like her sister, she showed developmental delay by the second year of age. After therapeutic intervention she began walking by the end of the second year. She also showed hyperactivity, agitation, aggressive behavior such as self‐injury as well as ID and ADHD. At the time of examination, she had normal head circumference of 47.5 cm. Her nonverbal IQ was 35–40 (severe impairment). Her cognition was severely delayed and she was able to say a few words. Behavior and social evaluation showed that she had severe deficit in adaptive behaviors in all domains including communication, daily living skills, and socialization and she was not able to perform any of her activities of daily living independently. Her motor development was normal, and her neurological examination was normal too. Brain magnetic resonance imaging (MRI) showed a thin but fully formed corpus callosum (Figure 1b) with no abnormalities involving the cerebral cortical gray matter. MRI also indicated near symmetrical signal abnormality in the bilateral external capsule, periventricular white matter, and abnormal T2 W hyperintensity. The height and weight were within the normal range as compared to the local population. No history of seizures or regression has been reported and there were no autistic features as well. Routine blood hematology and biochemistry tests were within the normal range for the patient.

### 1.4 Patient 3

**FIGURE 2:**
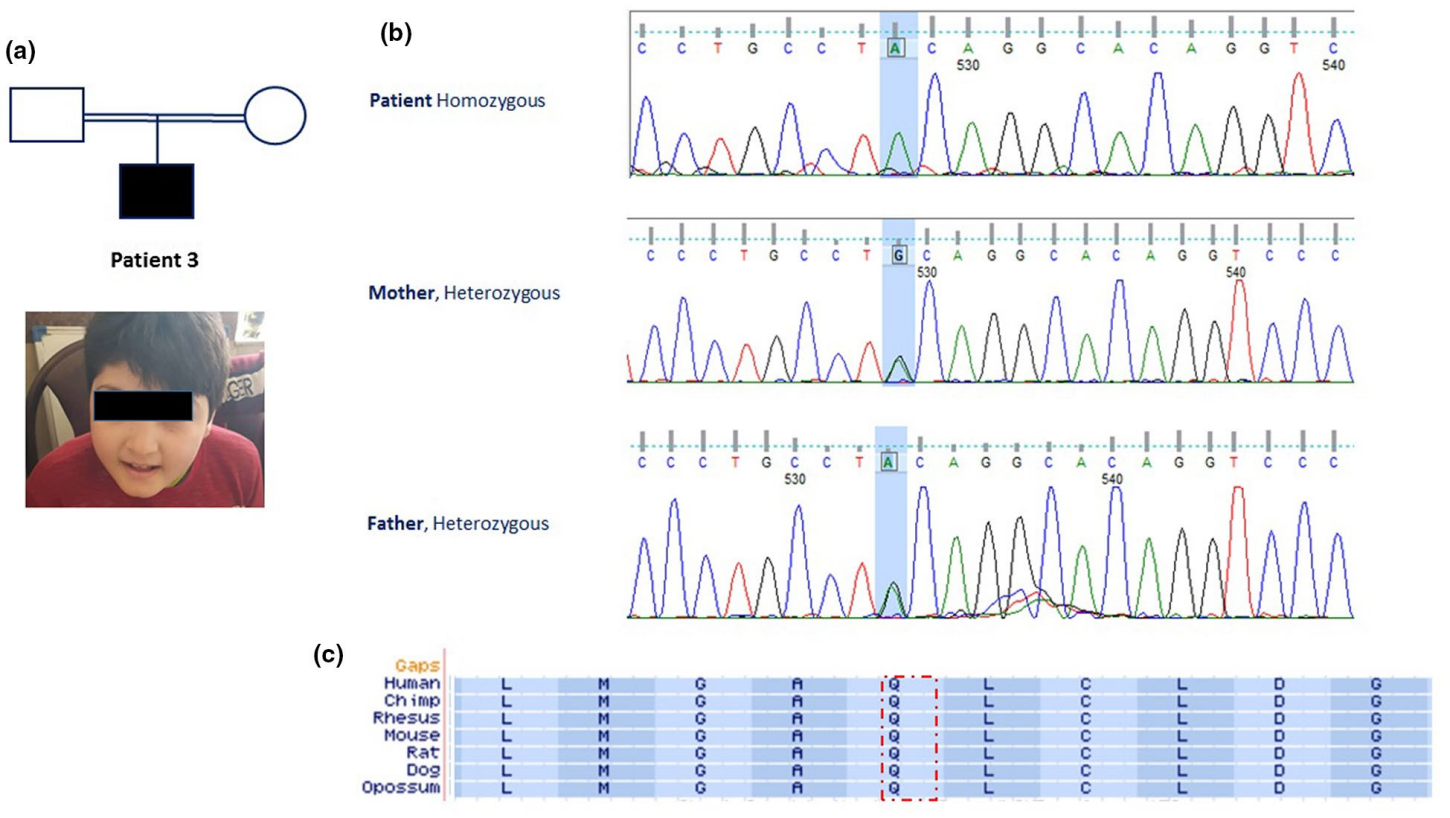
(Analysis of family 2; a) Pedigree of the consanguineous family 2 with one patient affected by intellectual disability with microcephaly. (b) Electropherograms from Sanger confirmation in family members showing NM_031466.7 (*TRAPPC9*: c.3103C>T; p.Arg1035Ter.) Heterozygous and homozygous sequence. (c) ClustalW2 (http://www.ebi.ac.uk/Tools/msa/clustalw2) alignment/ comparison of *TRAPPC9* across vertebrate species showing conservation at p.Arg1035 (red rectangle).

The patient (Figure 2) in family 2 was a 9‐year‐old boy and the first child of healthy consanguineous parents who was born via normal vaginal delivery within normal growth parameters after an unremarkable pregnancy and delivery. The birth head circumference was 33 cm (50th percentile). The weight at birth was 3 kg (15th percentile) and the birth height was 47 cm (15th percentile). His motor development was normal and he started walking at 12 months of age. He was found to have microcephaly (<3rd percentile) at age 1. Speech delay was noted thereafter. He was diagnosed with autism spectrum disorders (ASD) and ADHD at age 5. Aggressive behaviors such as self‐injury were also reported. At the time of examination, his OFC was 49 cm (−2 SD), his height was 142 cm (>90 percentile), and his weight was 52 kg (>97 percentile). His motor development and neurological examination were normal. He had severe cognitive delay and moderate speech delay. Behavior and social evaluation showed that he had severe deficit in adaptive behaviors in all domains including communication, daily living skills, and socialization and he was not able to perform any of his activities of daily living independently. As with the two other reported patients, he also showed agitation and restlessness. No history of seizure was reported. MRI was also performed for this patient which led to no clinically significant finding. Routine blood hematology and biochemistry tests were within the normal range.

## 2 METHODS

### 2.1 Ethical compliance

This study has been approved by the ethics committee of the National Institute of Genetic Engineering and Biotechnology (NIGEB) of Iran. Appropriate written informed consent for genomic analysis was obtained from parents.

Peripheral blood karyotype and multiplex ligation‐dependent probe amplification (MLPA) test using both syndromic microdeletion/duplication and subtelomeric microdeletion/duplication for both patients in family 1 and array CGH for the patient in family 2 were performed, which led to no clinically significant findings. To determine the molecular etiology, whole‐exome sequencing (WES) was performed. DNA was extracted from peripheral blood leukocytes of patient 1 in family 2 and patient 2 in family 2 using a commercial kit (High Pure PCR Template Preparation, Roche). Whole‐exome sequencing on DNAs was enriched for exonic regions with SureSelect 38 Mbp. All exon kit v. 7.0 (Agilent Technologies) was prepared according to manufacturer protocols, and 75 × 2 bp paired‐end sequenced on HiSeq 2000 (Illumina Inc.), with a 80–120× mean coverage. The sequencing quality was confirmed using FastQC 11.5 software (Bittencourt a, 2010). Variants were filtered in the preliminary whole exome data analysis was performed through Burrows–Wheeler Aligner (BWA; Li & Durbin, 2009) and the Genome Analysis Toolkit (GATK) software (McKenna et al., 2010) to generate a Binary Alignment Map (BAM) and a Variant Call Format (VCF) file, respectively. Annotations of the VCF files were carried out through the wANNOVAR software (Wang et al., 2010), and the data were manually analyzed for the presence of candidate pathogenic variants. Variants were filtered out in different human population databases. The local NGS database (www.iranome.ir), as well as public databases (the 1000 Genomes Project [Consortium, 2015]), the Genome Aggregation Database (gnomAD), the Genome Aggregation Consortium (ExAC; Lek et al., 2016), ESP6500 (Fu et al., 2013), and dbSNP 137 were also investigated. Pathogenicity of the variants were assayed using prediction methods (PolyPhen‐2 (Adzhubei et al., 2013), SIFT (Ng & Henikoff, 2003), MutationTaster (Schwarz et al., 2014), PROVEAN (Choi & Chan, 2015), and in silico nucleotide conservation from Genomic Evolutionary Rate Profiling (GERP) scores (Pollard et al., 2010)). VarSome database, a search engine for human genomic variation, was also used for classifying candidate variants according to the criteria set by the American College of Medical Genetics (ACMG; Richards et al., 2015) The identified mutations were validated using Sanger sequencing. The online version of Primer 3 software was used to design primers flanking candidate variants.

Regions were amplified and sequenced using ABI 3500 Genetic Analyzer (Applied Biosystems Inc., 850 Lincoln Center Drive). Segregation analysis using Sanger sequencing was performed for parents in both families and patient 2 in family 1 at the mutations’ positions.

## 3 RESULTS

**FIGURE 3:**
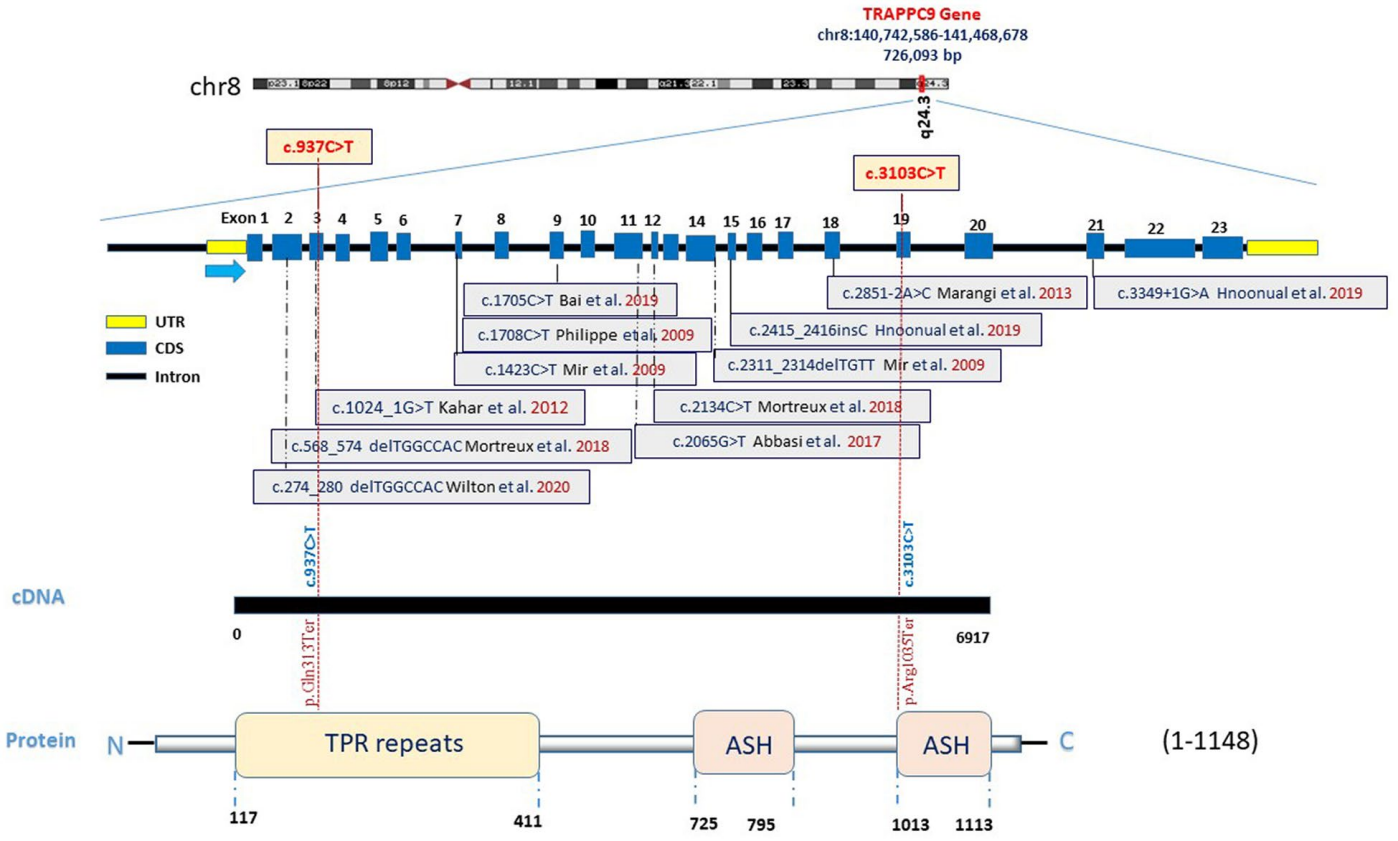
*TRAPPC9* (NG_016478.2, NM_031466.7) structure and mutation positions at cDNA and protein level.

In patient 1 from family 1, a novel homozygous nonsense mutation (c.937C>T) in exon 3 and in the patient from family 2, a novel homozygous nonsense mutation (c.3103C>T) in exon 19 of *TRAPPC9* (NG_016478.2, NM_031466.7; Figure 3) were identified. The c.937C>T mutation changes Glutamine in position 313 to stop codon (p.Gln313*). While the c.3103C>T mutation creates a stop codon by changing Arginine in position 1035 (p.Arg1035*). Sanger sequencing confirmed heterozygosity for parents in both families and homozygosity for patient 2 in family 1 mutations’ positions (Figures 1c and 2b).

The forward and reverse primers for c.3103C>T and c.937C>T are as follows, respectively:

F (exon3) _AAAACGACTCCAGATTCTCCCA, R_TGTTCAGAACCAGTCTCCGTAAT F (exon19)_: CACAGGCTGGCTGCTTTTCCT, R_AGACCACTCCAGCTCACATAACT.

The variants are not present in population databases Iranom, the 1000 Genomes Project (Consortium, 2015), the Genome Aggregation Database (gnomAD), the Genome Aggregation Consortium (ExAC; Lek et al., 2016), ESP6500, and dbSNP.

Detailed computational analysis of p.Gln313Ter and p.Arg1035Ter mutations using prediction methods (PolyPhen‐2, SIFT, MutationTaster, and PROVEAN revealed both mutations as disease‐causing. According to the ACMG criteria, both variants are considered to be pathogenic.

## 4 DISCUSSION

Here, we describe three Iranian patients with ID, born to two consanguineous families of Persian descent and report two novel homozygous alterations in TRAPPC9 gene. These mutations in exon 3 (c.937C>T) and exon 19 (c.3103C>T) change amino acids in positions 313 and 1035 to stop codon, respectively, resulting in a premature termination and a truncated protein. A homozygous splicing mutation in exon 3 of *TRAPPC9* gene has been reported recently (Hnoonual et al., 2019; Kakar et al., 2012), but mutation in exon 3 of the patient in family 2 is a novel homozygous nonsense mutation. Moreover, the nonsense mutation in *TRAPPC9* gene of our patients in family 1 resides in exon 19 in which no other pathogenic alterations have yet been reported.

*TRAPPC9* contains 23 exons, coding for 1148 amino acid, and encodes the NIK‐ and IKK‐beta binding protein (NIBP), which is highly conserved across evolution. From the multiple NIBP isoforms, only isoform 1 is present in the brain, where it is expressed in the cell bodies and processes of neurons. The function of the NIBP protein domains has not yet been determined. However, a direct interaction between NIBP with NIK and IKK‐beta has been reported to be involved in both classical and alternative NF‐kB signaling pathways, which may involve neuronal processes, including neuronal cells differentiation, synaptic plasticity, and neurogenesis (Denis‐Donini et al., 2008; Hu et al., 2005).

The loss of the NIBP function has been reported to reduce TNFa‐induced NF‐kB activation prevent nerve growth factor‐induced neuronal differentiation (Marangi et al., 2013). This led to the notion that the loss of the NIBP function might cause disruption of neuronal differentiation, axon growth, delayed myelination of the white matter, and cerebral white matter hypoplasia (Marangi et al., 2013; Mir et al., 2009). Loss of TRAPPC9 has been suggested to play a role in apoptotic signal in neurons leading to an increased cell loss during development, which resembles the clinical hallmark of microcephaly (Abbasi et al., 2017). The homozygous c.937C>T and c.3103C>T variants occur in the evolutionary conserved regions of NIBP protein (Figures 1d and 2c), highlighting the role of NIBP domain in neuronal processes, including neuronal cell differentiation, synaptic plasticity, and neurogenesis may lead to manifestations in patients of our study. To date, different *TRAPPC9* mutations (homozygous and compound heterozygous mutations, homozygous and compound heterozygous for copy number variation [CNVs]) have been reported in unrelated families from various origins (Abbasi et al., 2017; Jamra et al., 2011; Bai & Kong, 2019; Giorgio et al., 2016; Hnoonual et al., 2019; Kakar et al., 2012; Koifman et al., 2010; Marangi et al., 2013; Mir et al., 2009; Mochida et al., 2009; Mortreux et al., 2018; Philippe et al., 2009; Wilton et al., 2020; Figure 3) which all lead to the loss of the TRAPPC9 protein. Mutations in *TRAPPC9* gene are ubiquitously scattered throughout the gene. This means that there should be no mutation hotspot in *TRAPPC9* gene and that every single exon plays a key role in maintaining normal function of the TRAPPC9 protein.

Clinical manifestations of mutations associated with *TRAPPC9* include ID, developmental delay, microcephaly, brain abnormalities, dysmorphic facial features, and obesity have been reported in previous studies (Table 1).

**TABLE 1:** Comparison of available clinical features between previous case reports with *TRAPPC9* mutations and patients in this study.

Clinical features	Present study			Previous reports	Total # (Including our patients)
	Patient 1, family 1	Patient 2, family 1	Patient in family 2		
Developmental delay	+	+	+	44/44	47/47 (100%)
Autistic features	−	−	+	5/20	6/23 (26%)
Microcephaly	−	−	+	40/42	41/45(91.1%)
Obesity	−	−	−	12/24	12/27 (44.4%)
Seizure	−	−	−	5/32	5/35 (14.2%)
Brain abnormality					
Thin corpus callosum	+	+	−	18/18	20/21 (95.2%)
Cerebral hypoplasia	+	+	−	12/12	14/15(93.3%)
Cerebellar hypoplasia	−	−	−	8/11	8/14 (57.1%)
Abnormality signal of white matter	+	+	−	18/19	20/22 (90.9%)
Dysmorphic features	−	−	−	22/37	22/40(55%)

Patients in both families have ID and developmental delay (DD), which is in line with previous reports of patients with *TRAPPC9* homozygous mutations.

Microcephaly is a clinical feature that has been reported in the majority of patients with homozygote mutations in *TRAPPC9*, (Table 1). The patients presented in family 1 of our study show normal head circumference as compared to the matched mean for age and sex.

In the cases previously reported, two patients did not show microcephaly: a patient from Pakistan (Mir et al., 2009) and another one from Italy (Mortreux et al., 2018). There are similarities in terms of clinical features between them and the patients in our study, which include ID, motor delay, speech delay, normocephalic, and MRI findings. Dysmorphic feature in Italian cases is the only difference between the patients (Mortreux et al., 2018). The patient in family 2 exhibited microcephaly, which is in line with reports of cases with mutations in this gene. Autistic features are a relatively rare phenomenon in patients with *TRAPPC9* mutations (Table 1). Unlike the patients in family 1, the patient in family 2 of our study demonstrated autistic features as well.

Furthermore, two cases with *TRAPPC9* mutations were recently reported with autistic features as well as ID (Hnoonual et al., 2019; Wilton et al., 2020), supporting the idea that *TRAPPC9* mutation may lead to heterogeneous clinical phenotypes. Our patient and the patients in the cited two studies showed ID, developmental delay, and microcephaly. However, unlike these cases, our patient has normal MRI and did not display dysmorphic facial features.

It is of note to mention that patients in both families of our study presented with ADHD. Interestingly, while patient 1 in family 1 presented with flexion contracture of elbow and fingers as well as agitation and restlessness, her sibling did show the latter but not the former. This may be explained, at least to some extent, by the idea that mutations in *TRAPPC9* cause a heterogeneous clinical phenotype, further accompanied by inter‐ and intra‐familiar differences.

In conclusion, we report three patients diagnosed with ID carrying two novel homozygous nonsense mutations in *TRAPPC9*. Additional reports from a large cohort of cases with *TRAPPC9* mutations are required to clarify certain phenotype–genotype correlation in *TRAPPC9*‐related disorder.

## AUTHORS’ CONTRIBUTIONS

Farideh Yousefipour proposed and designed the concept, collected and analyzed the data, and wrote the manuscript. Hossein Mozhdehipanah voluntarily examined the patients. Frouzandeh Mahjoubi had responsibility of experiments; interpreted and analyzed the results, revised the manuscript and supervised the work. All the authors read and approved the final manuscript.

## Supporting information

Table S1

## Data Availability

The data that supports the findings of this study are available in the supplementary material of this article.
