# Supplementary material for: Identification of two novel homozygous nonsense mutations in TRAPPC9 in two unrelated consanguineous families with intellectual Disability from Iran
Source: Mol Genet Genomic Med. 2021 Jan 29;9(12):e1610. doi: 10.1002/mgg3.1610 (PMC8683625; doi:10.1002/mgg3.1610)
Supplement: Supplementary file 1 — Table S1 [file MGG3-9-e1610-s001.docx]

**Supplementary Table S1**

Clinical features of patients with TRAPPC9 mutations from the literature review.

| **Clinical features** | **Homozygous mutations, Homozygous deletion/duplication** | | | | | | | | | | | | | | | **Compound**  **heterozygous CNV +**  **rare variant** | | |  | **Total previous case reports** |
| --- | --- | --- | --- | --- | --- | --- | --- | --- | --- | --- | --- | --- | --- | --- | --- | --- | --- | --- | --- | --- |
|  | **Mir et al., 2009** | | | **Mochida et al., 2009** | **Philippe et al., 2009** | **Koifman et al., 2010** | **Abou Jamra et al., 2011** | **Kakar et al., 2012** | **Marangi et al., 2013** | | **Giorgio et al., 2016** | **Abbasi et al., 2017** | | **Mortreux et al., 2018** | | | | Hnoonual et al.,  2019 | Wiltom et al.,  2020 |  |
| Origin | Pakistan | Iranian | | \|  \| Israeli Arab \| \| --- \| --- \| | Tunisian | Filipino | Syrian | Pakistan | Italian | | Egyptian | Pakistan | Pakistan | Algerian | Tunisian | Italian | French | Thai | Malta |  |
| Consanguinity | Yes | Yes | | Yes | Yes | Yes | Yes | Yes | Yes | | Yes | Yes | Yes | Yes | Yes | No | No | No | Yes |  |
| No. Affected individuals | 7 | 3 | | 3 | 3 | 1 | 6 | 3 | 2 | | 1 | 3 | 3 | 1 | 3 | 1 | 1 | **2** | **1** | **44** |
| Male: Female | 1:6 | 3:0 | | 0:3 | 3:0 | 0:1 | 3:3 | 0:3 | 0:2 | | 0:1 | 2:1 | 3:0 | 0:1 | 1:2 | 0:1 | 0:1 | 1:1 | 0:1 | **18:26** |
| Diagnosis | **ID** | **ID** | | **ID** | **ID** | **ID** | **ID** | **ID** | **ID** | | **ID** | **ID** | **ID** | **ID** | **ID** | **ID** | **ID** | **ID** | **ID** | **ID** |
| *TRAPPC9*  mutation | c.1423C>T (p.Arg475*) | c.2311_2314  delTGTT  (p.Leu772Trpfs*7) | | c.1423C>T (p.Arg475*) | c.1708C>T (p.Arg570*) | 141.46 kb  deletion of 8q24.3  including  *TRAPPC9* | c.1423C>T (p.Arg475*) | c.1024+1G>T | c.2851-2A>C (p.Thr951Tyrfs*17) | | c.1423C>T (p.Arg475*) | c.2065G>T (p.Glu689*) | c.1423C>T (p.Arg475*) | 115 kb duplication in  *TRAPPC9a* | c.1708C>T (p.Arg570*) | 119 kb duplication in  *TRAPPC9*  +  deletion variant^b^ | 189 kb deletion in *TRAPPC9*  +  c.2134C>T, (p.Arg712*) | c.2415-2416 insC; c.3349+1G->A | c.568-574del |  |
| Developmental delay | 7/7 | 3/3 | | 3/3 | 3/3 | 1/1 | 6/6 | 3/3 | 2/2 | | 1/1 | 3/3 | 3/3 | 1/1 | \| 1/1 \| 3/3 \| \| --- \| --- \| | 1/1 | 1/1 | 2/2 | 1/1 | **44/44 (100%)** |
| Autistic features | 0/3 | NA | | 0/3 | NA | NA | NA | 0/3 | 0/2 | | NA | NA | NA | 0/1 | 1/3 | 1/1 | 1/1 | 1/2 | 1/1 | **5/20 (25%)** |
| Microcephaly | 5/6 | 3/3 | | 3/3 | 3/3 | 1/1 | 6/6 | 3/3 | 2/2 | | 1/1 | 2/2 | 3/3 | 1/1 | 3/3 | 0/1 | 1/1 | 2/2 | 1/1 | **40/42 (95.2%)** |
| Obesity | NA | NA | | NA | 3/3 | NA | NA | 0/3 | 2/2 | | 1/1 | 0/3 | 0/3 | 0/1 | 3/3 | 1/1 | 0/1 | 2/2 | 0/1 | **12/24 (50%)** |
| Seizure | 1/3 | 0/3 | | NA | NA | NA | 1/6 | 0/3 | 1/2 | | NA | 1/3 | 1/3 | 0/1 | 0/3 | 0/1 | 0/1 | 0/2 | 0/1 | **5/32 (15.6%)** |
| **Brain abnormalities** |  | | | | | | | | | | | | | | | | | | | |
| Thin corpus callosum | 3/3 | | NA | 2/2 | NA | 1/1 | NA | NA | | 2/2 | 1/1 | NA | NA | 1/1 | 3/3 | 1/1 | 1/1 | 2/2 | 1/1 | **18/18 (100%)** |
| Cerebral hypoplasia | 3/3 | | NA | 2/2 | NA | 1/1 | NA | NA | | 2/2 | 1/1 | NA | NA | NA | NA | NA | NA | 2/2 | 1/1 | **12/12 (100%)** |
| Cerebellar hypoplasia | 3/3 | | NA | 1/1 | NA | 1/1 | NA | NA | | 2/2 | 0/1 | NA | NA | NA | NA | NA | NA | 0/2 | 1/1 | **8/11 (72.7%)** |
| Abnormal signal of white matter | 3/3 | | NA | 0/1 | 2/2 | 1/1 | NA | NA | | 2/2 | 1/1 | NA | NA | 1/1 | 3/3 | 1/1 | 1/1 | 2/2 | 1/1 | **18/19 (94.7%)** |
| Dysmorphic facial featuresd | 0/3 | | 0/3 | NA | 2/3 | 1/1 | 6/6 | 0/3 | | 2/2 | 1/1 | 1/3 | 0/3 | 1/1 | 3/3 | 1/1 | 1/1 | 2/2 | 1/1 | **22/37 (59.4%)** |

NA, not available; ID, intellectual disability;
